# The Bushland, Texas, alfalfa, soybean, sunflower, and winter wheat evapotranspiration, growth, and yield dataset collections

**DOI:** 10.1038/s41597-025-06225-9

**Published:** 2025-12-17

**Authors:** Steven R. Evett, Gary W. Marek, Paul D. Colaizzi, Karen S. Copeland, Brice B. Ruthardt

**Affiliations:** USDA Agricultural Research Service, Conservation & Production Research Laboratory, Bushland, Texas USA

**Keywords:** Environmental sciences, Plant sciences

## Abstract

Datasets are presented for experiments on alfalfa (*Medicago sativa* L.) (1996–1999), soybean (*Glycine max* (L.) Merr.) (1995, 2003, 2004, 2010, 2019), sunflower (*Helianthus annuus* L.) (2009, 2011), and winter wheat (*Triticum aestivum*, L.) (1989–1990, 1991–1992, 1992–1993). Weighing lysimeters were used to determine crop evapotranspiration (ET). In-soil and above ground microclimate and ET data are presented on a 15-minute interval as are weather data for all days of the year. Soil water content data from calibrated neutron probe readings are presented on a periodic basis. Crop planting, harvest, fertilization, pest control, and other agronomic information for all 12 seasons are presented in agronomic calendars by day of year. Crop growth data are presented on a periodic basis throughout the growing season, as are crop biomass and yield data for each harvest. The data are suitable for model calibration, testing, and improvement, and for analysis of effects of weather, irrigation and other agronomic decisions on crop yield and water productivity in the Southern High Plains region of the USA.

## Background & Summary

**Fig. 1:**
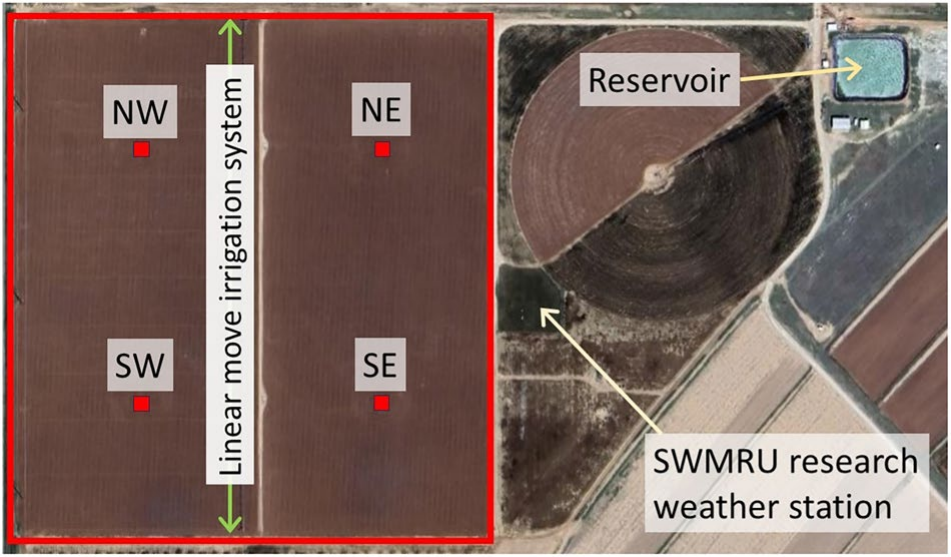
USDA ARS Bushland, Texas, irrigation, weighing lysimeter, weather, and related facilities in 2012. The four fields are arranged in a square, outlined in red in the illustration. The red squares indicate positions of the lysimeters.

As reported in previous works^1,2^, in 1987 and 1988, four large, precision weighing lysimeters were built by USDA ARS at Bushland, Texas, to measure evapotranspiration (ET)^2^ by mass balance (Fig. 1). The research purpose was to determine the effects on ET of crop choice, variety, agronomic practices (tillage, applications of fertilizers and pesticides, etc.), irrigation management, and irrigation application method^3^ for the major crops grown on the southern High Plains. Purposes originally included providing regionally specific crop coefficients^4–6^ for irrigation management that would be used in a regional scheduling network, developing and improving crop simulation models by determining the energy and water balances of growing crops^7,8^, developing and testing other methods for determining ET, and developing quality-controlled datasets for crop model testing, improvement, and intercomparison^9^. For these purposes, not only ET data but energy and water flux, plant growth and yield, soil water content^10^ and temperature, and weather^11^ data were collected, and methods for quality control of all data were developed^12,13^. This report describes the organization and details of four collections of datasets: One for four years when alfalfa^14^ (*Medicago sativa* L.) was grown as a reference ET^15^ crop and for hay; one for five years when soybean^16^ (*Glycine max* (L.) Merr.) was grown for seed grain; one for two years when sunflower^17^ (*Helianthus annuus* L.) was grown for seed, and one for three seasons when winter wheat^18^ (*Triticum aestivum*, L.) was grown for grain.

## Methods

All experiments took place at the USDA-ARS Conservation and Production Laboratory (CPRL), Soil and Water Management Research Unit (SWMRU), Bushland, Texas USA (Lat. 35.186714°, Long. −102.094189°, elevation 1170 m above MSL) on the Pullman soil series, a fine, mixed, superactive, thermic Torrertic Paleustoll. There are 1.54 million hectares of Pullman soil on the Southern High Plains, much of it in row crops, both irrigated and dryland. Crops were grown on from two to four of the four large, precision weighing lysimeters and their surrounding fields. Each lysimeter was in the center of a 4.44 ha square field. As described elsewhere^1,2^, each lysimeter consisted of a monolithic, undisturbed, rectangular prism of soil measuring 3 m by 3 m horizontally and ~2.3-m deep. Cores were taken on site in steel containers measuring 2.4-m in depth, allowing for a vacuum drainage system installed in a 0.10-m deep layer of fine sand at the bottom. In the lysimeters and nearby field areas that could not be reached with regular farm machinery, tillage was performed by hand and using hand-guided small machines to the same depth as in the surrounding fields and at the same times as for field tillage. Pesticide and herbicide applications were performed at the same rates and times as done in the field. Amounts and kinds of fertilizer applied were based on soil tests early in each year. The four fields were contiguous and arranged in four quadrants, which were labeled northeast (NE), southeast (SE), northwest (NW), and southwest (SW) (Fig. 1). See the resource titled “Geographic Coordinates, USDA, ARS, Bushland, Texas” for UTM geographic coordinates of field and lysimeter locations. The land slope is <1% and topography is flat. The mean annual precipitation is ~470 mm, the 20-year pan evaporation record indicates ~2,600 mm Class A pan evaporation per year, and winds are typically from the South and Southwest. The climate is semi-arid with ~70% (350 mm) of the annual precipitation occurring from May to September, during which period the pan evaporation averages ~1520 mm. Irrigation was by a 10-span linear move irrigation system on all fields until 2013 when a subsurface drip irrigation (SDI) system was installed in the NE and SE fields and lysimeters. The SDI system had drip tape lines spaced at 1.52 m in the middle of every other interrow and buried at 0.30 to 0.32 m^19^.

### Soil water sensing and irrigation management

Soil water content readings were made with a field calibrated neutron probe from 0.10- to 2.40-m depth in 0.20-m depth increments in the field. Readings in the two access tubes in each lysimeter were made at depths from 0.10- to 1.90-m, again at 0.20-m depth increments. The number and spacing of neutron probe reading field locations changed through the years (additional sites were added), which is one reason why subsidiary datasets and data dictionaries are needed to specify those locations. Resulting soil water content data are in the dataset, “Soil Water Content Data for The Bushland, Texas Large Weighing Lysimeter Experiments”^10^. Fertilizer and pesticide applications were the same across fields and lysimeters. Irrigations designated as less than full (i.e. Deficit) were applied as a percentage of the full irrigation amount. Irrigations designated as “full” were managed to replenish soil water used by the crop on a weekly or more frequent basis as determined by soil water content readings.

### Crops grown

Alfalfa was planted in fall 1995 and grown on two (NE and SE) of the weighing lysimeters and fields. Irrigation was by linear move sprinkler system using mid elevation spray applicators (MESA) positioned at 1.5-m above the soil surface and spaced 1.52-m apart along the irrigation lateral. Irrigations were “full” in 1996, 1997, 1998, and the first part of 1999 after which some deficit irrigations were applied. Daily logs were kept of all field operations (tillage, fertilizer and pesticide applications, planting, plant measurements, harvest, etc.), lysimeter maintenance and hand tillage, abnormal weather events, and manual measurements that could impact lysimeter readings. These logs are in one spreadsheet for all years, including the fall of 1995, which is a resource available as file “1995–1999 Alfalfa Calendar.xlsx”, “Agronomic Calendar for the Bushland, Texas Alfalfa Datasets”.

**Fig. 2:**
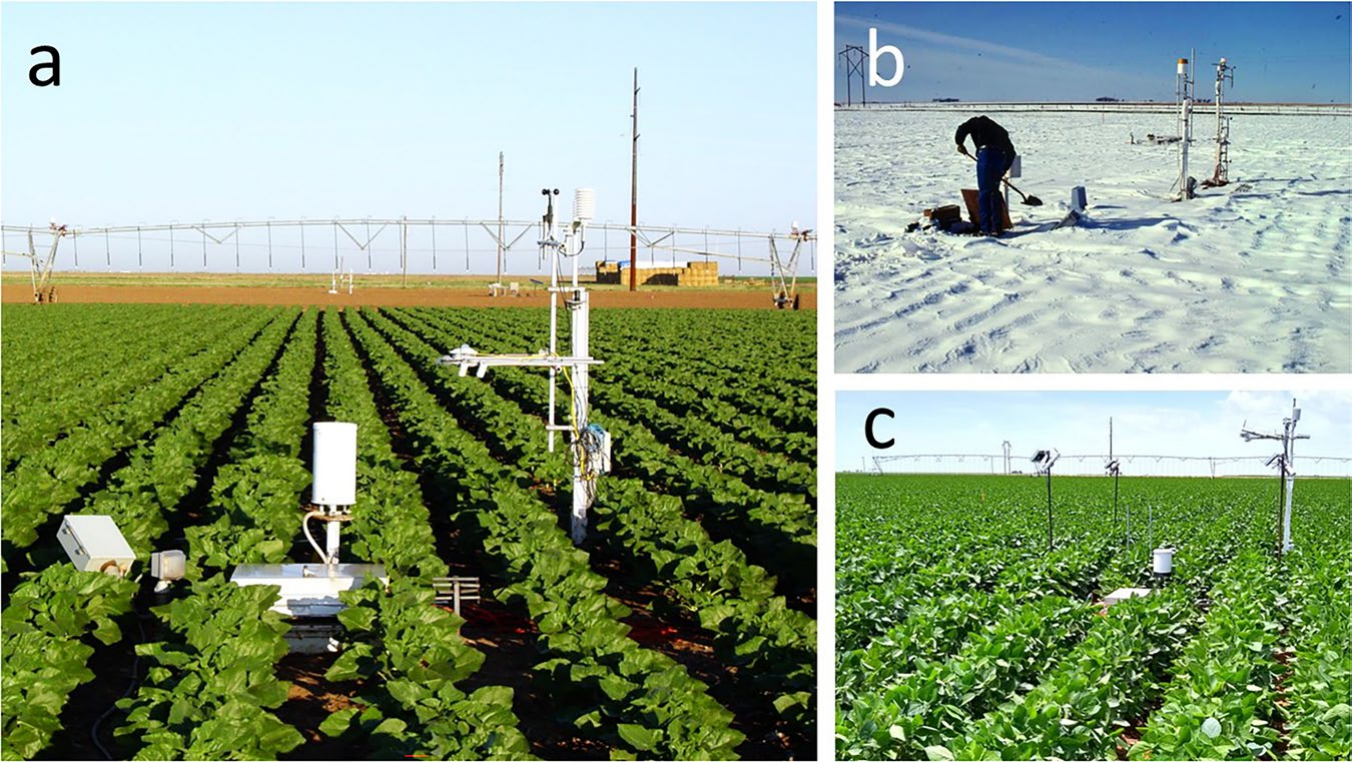
Photos of some of the crops on a weighing lysimeter showing relative positions of access hatch, rain gauge, and micrometeorological masts. (**a**) Sunflower crop. The rain gauge and access hatch are shown in the foreground, and the mast carrying micrometeorolgical instrumentation is shown on the right (north) side of the lysimeter, which itself is not visible. The linear move irrigation system is visible in the background. (**b**) Snow covering winter wheat on a lysimeter. Snow is being shoveled off the hatch so that the lysimeter inside instrumentation can be accessed. (**c**) A soybean crop on a weighing lysimeter. Masts carrying micrometeorological instrumentation, including infrared thermometers are visible at the corners of the lysimeter.

In 1995 and 2010, soybean was grown on only the NW and SW fields and lysimeters (Fig. 2). Soybean was grown on only the NE and SE fields and lysimeters in 2003 and 2004. Irrigation was by linear move sprinkler system, and that system was equipped with mid-elevation spray applicators (MESA). In 2019, soybean was grown on all four large, precision weighing lysimeters, and on the fields surrounding each lysimeter. The NE and SE fields and lysimeters were irrigated using subsurface drip irrigation (SDI), and NW and SW fields and lysimeters were irrigated with the linear move sprinkler system. Daily logs are in the dataset, “Agronomic Calendars for the Bushland, Texas Soybean Datasets”^20^.

Sunflower was grown for seed on the NE and SE fields and lysimeters in both 2009 and 2011. Irrigation was by linear move sprinkler system equipped with mid-elevation spray applicators (MESA). Daily logs are in the dataset, “Agronomic Calendars for the Bushland, Texas Sunflower Datasets”^21^.

Winter wheat was grown on only the NW and SW fields for the 1989–1990 and 1992–1993 seasons, and on only the NE and SE fields in the 1991–1992 season. Irrigation was by linear move sprinkler system, and that system was equipped with various application technologies such as high-pressure impact sprinklers, low pressure spray applicators, and low energy precision applicators (LEPA). Daily logs are in the dataset, “Agronomic Calendars for the Bushland, Texas Winter Wheat Datasets”^22^.

### Weighing lysimeter measurement and instrumentation

Weighing lysimeter calibration confirmed accuracy of 0.05 mm or better for data acquired at 5-minute intervals (mean of 300 readings taken on 6-s intervals). Calibration used masses traceable to NIST^23^. Lysimeters were used to measure mass, which was converted to relative soil water storage in mm depth of water considering the density of water and the surface area of the lysimeter. Equation 1 presents the soil water balance for calculation of ET in which the change in soil water storage (∆*S*, mm), the precipitation (*P*, mm), the dew and frost accumulation (DW, mm), the irrigation amount (*I*, mm), the sum of runon and runoff (*R*, mm), and the subsurface soil water flux into or out of the soil control volume (*F*, mm) are summed to calculate crop evapotranspiration (ET).

1 $${\rm{ET}}=\varDelta S+P+{\rm{DW}}+I+F+R$$

A weighing lysimeter represents a special case of a soil control volume in which *F* is controlled. Fields were managed to reduce runon and runoff so that in most cases *R* in fields was negligible. The soil monolith container freeboard was approximately 50 mm so that *R* for a lysimeter was negligible except during infrequent very large precipitation events. In the rare event that *R* was not negligible, data were flagged to indicate that ET data were unreliable. The lysimeters were equipped with vacuum drainage systems operating at 1-m water head of suction. Drainage was captured in tanks suspended from the lysimeter monolith such that drainage into the tanks did not change the mass of the lysimeter, meaning that although drainage into the tanks occurred, the value of *F* in Eq. 2 was zero. Tanks were suspended from load cells, the output of which was recorded as drainage into each tank. Because drainage into the tanks did not change lysimeter mass, the recorded drainage into the tanks was not used in calculating ET using Eq. 2. Drainage under the conditions of these studies was small enough that tanks sometimes did not fill during a year. When tanks did fill they were emptied and the change in mass recorded by the lysimeter weighing system was converted to an equivalent depth of water, resulting in a negative *F* value for Eq. 2 during the period of tank emptying, which typically was 15 minutes or less.

In addition to the components of the soil water balance shown in Eq. 1, a weighing lysimeter system can include virtual components (Eq. 2). These include any additions to or subtractions from the lysimeter mass that are not due to water balance components. For example, scale counterweight adjustments (CW) made to keep the scale load cell within its operational range cause changes in recorded relative water storage. Occasionally, other lysimeter mass changes occur due to operations and maintenance and must be accounted for. These are indicated by a “V” flag. Including the virtual components, the water balance equation becomes.

2 $${\rm{ET}}=\varDelta {\rm{S}}+{\rm{P}}+{\rm{DW}}+{\rm{I}}+{\rm{F}}+{\rm{R}}+{\rm{CW}}+{\rm{V}}$$

Amounts of ET, change in storage, irrigation, precipitation and dew and frost accumulation were determined by applying Eq. 2 to changes in relative water storage using a spreadsheet developed for this purpose^24,25^.

A suite of micrometeorological instruments was installed at each lysimeter to sense precipitation, wind speed, air temperature and humidity, radiant energy (incoming and reflected, typically both shortwave and longwave), surface temperature, soil heat flux, and soil temperature, all of which are reported at 15-minute intervals. Instruments used changed from season to season, which is another reason that subsidiary datasets and data dictionaries for each season are required. In some years, radiant energy at the soil surface was sensed, as well as photosynthetically active radiation (PAR). Tipping bucket rain gauges on fixed-height masts were used at each lysimeter to sense precipitation, and data from them are questionable when crop height was greater than the gauge orifice height. For this and other reasons discussed in the section on Weather data, precipitation data from gauges at the lysimeters are considered only indicative of the occurrence of precipitation and sprinkler irrigation, not definitive of amounts of these. The data dictionaries describe the instrument used to sense each environmental parameter in each year. Important conventions concerning the data-time correspondence, sign conventions, and terminology specific to the USDA ARS, Bushland, TX, field operations are given in the resource titled “Conventions for Bushland, TX, Weighing Lysimeter Datasets”.

A separate dataset was developed using more exacting and time-consuming processing of 5-minute lysimeter data. This more carefully quality controlled dataset contains 15-minute and daily (midnight to midnight) mean values of evapotranspiration, irrigation, precipitation, dew and frost accumulation, and drainage tank emptying data. These data are considered the Bushland data of record for ET, irrigation, precipitation, and dew/frost accumulation amounts that would be used for simulation model calibration, testing, improvement, and intercomparison. These curated datasets are called the “Evapotranspiration, Irrigation, Dew/frost - Water Balance Data for The Bushland, Texas *CROP NAME* Datasets”, where *CROP NAME* is either “Alfalfa”, “Soybean”, “Sunflower”, or “Winter Wheat”.

### Plant growth and yield measurement

Plant emergence, growth, and yield measurements were made in multiple replicate plots in each field, and the number of replicates and sizes of plots did change from year to year and so are recorded in the data dictionaries for the datasets named “Growth and Yield Data for the Bushland, Texas *CROP NAME* Datasets”, where *CROP NAME* is either “Alfalfa”, “Soybean”, “Sunflower”, or “Winter Wheat”. Plant stand was measured during and through the completion of emergence to determine the density of plants in the machine-planted fields. On an approximately biweekly basis, as determined by weather and other circumstances that prevented entrance to the fields, growth stage, plant height, leaf area index, row width (in the case of row-planted crops), and above ground biomass were measured in replicate plots in each of the fields, while plant height was measured on the weighing lysimeters. Leaf area index and above-ground biomass were determined from destructive harvest of all above-ground biomass, which was put into tared plastic bags to prevent water loss, and weighed, then separated into leaves and stems and weighed again. Before drying, the leaves were run through a digital scanning bed leaf area meter (model LI-3100, LI-COR, Lincoln, Neb. USA), which was frequently calibrated using test disks, to determine total leaf area. Leaves and stems were dried at 60 °C until constant mass and then weighed again.

Both machine harvest in replicate measured areas in each field and hand harvest in replicate plots in each field were accomplished at each cutting of alfalfa and at crop maturity for soybean, sunflower, and winter wheat. For hand harvest, the entire above-ground biomass from each replicate plot in the field and from each lysimeter was harvested and separated into leaves, stems, pods in the case of soybean, and heads in the cases of sunflower and winter wheat, each of which were weighed then dried at 60 °C until constant mass, then weighed again. Alfalfa hay was baled (small rectangular bales), and multiple replicate subsamples were taken for forage quality analysis. Yields were reported as dry kg/ha. Wheat heads were threshed and grain number, total grain mass, and mass per grain were measured. Soybean pods and sunflower heads were threshed and seed grain number, total grain mass, and mass per grain were measured. For pulse, seed, and grain crops, harvest index was reported on a dry mass basis, and yields are reported as both dry grain Mg/ha and as bushels per acre at standard moisture content.

### Weather data

**Fig. 3:**
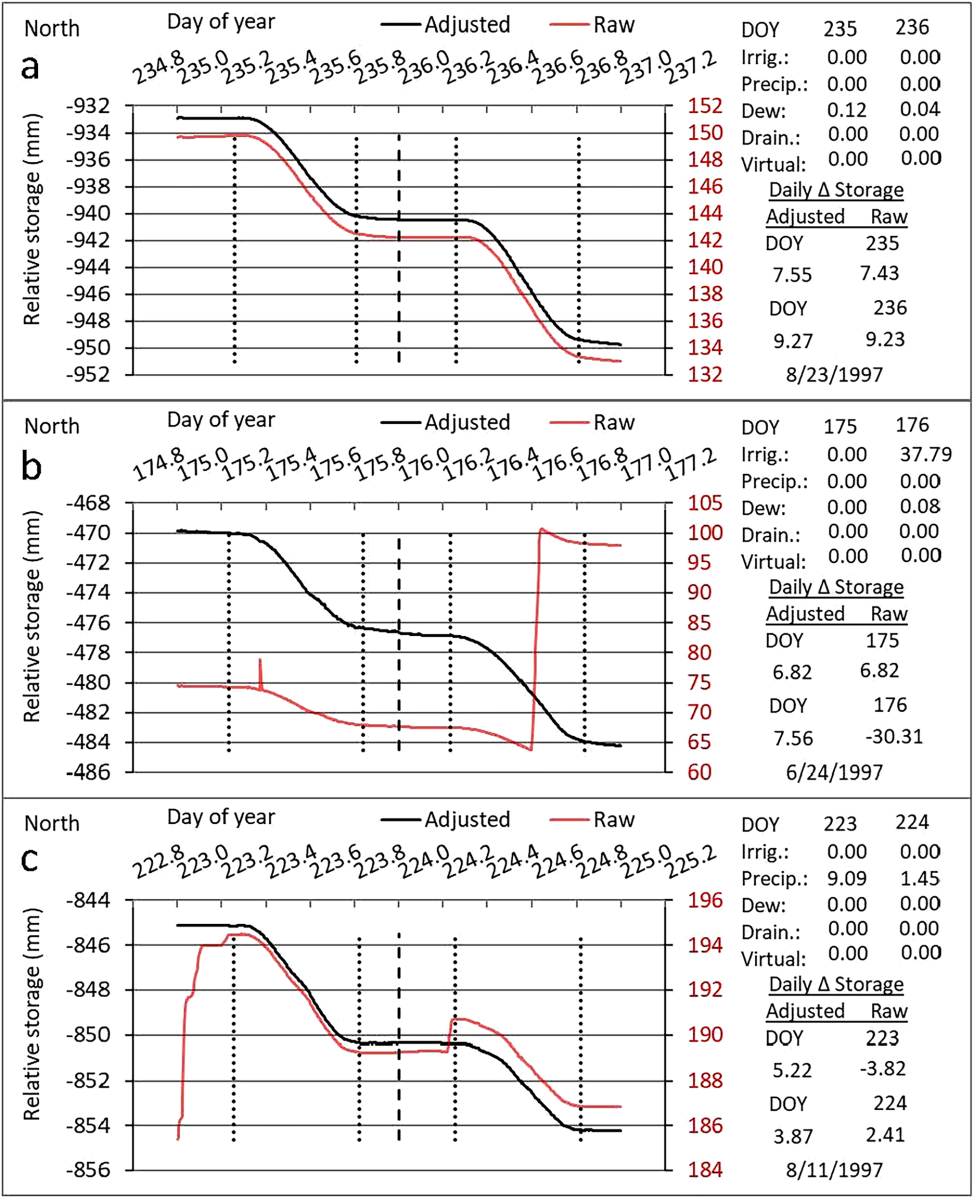
Illustrations of raw lysimeter data (red) in 1997 showing the change of relative storage (mass) over time due to various events, and adjusted data (black) after the events are taken into account such that relative storage change reflects only evapotranspiration. Vertical dotted lines indicate sunrise and sunset; vertical dashed line indicates midnight. (**a**) Change in relative storage over time on two days of year (DOY) when no events other than evapotranspiration and minor dewfall caused changes in lysimeter mass. (**b**) Illustration of raw lysimeter data with a spike due to neutron probe reading on the lysimeter on DOY 175, and an irrigation on DOY 176. (**c**) Change in relative storage over time when a rainfall event continued after midnight until just before dawn on DOY 223, and another rainfall occurred before dawn on DOY 224.

Adjacent to the NE and SE lysimeter fields (Fig. 1), weather data were gathered on a 0.4-ha flat weather station from replicate calibrated sensors at 2-m and 10-m heights over grass mowed to 0.12-m height. This was in addition to meteorological data sensed at each lysimeter. The weather station grass was irrigated by flood prior to 1994, and by subsurface drip irrigation in 1994 and subsequent years and managed to maintain a reference grass surface (well-watered and fertilized, cut to standard height). Solar irradiance was sensed with Eppley and LI-COR solar irradiance sensors. Wind speed and direction were sensed with wind speed and direction sensors at both 2-m and 10-m heights. Relative humidity and air temperature sensors^26^ were mounted in both a cotton belt shelter and at 2-m height on the mast. Precipitation was measured with a tipping bucket rain gauge with 0.20-m orifice and wind screen, later replaced with a heated tipping bucket rain gauge. Nonetheless, reported precipitation data were determined from weighing lysimeter mass increase during the event, converted to an equivalent depth of water. Precipitation data from the tipping bucket gauges at the weather station and at the lysimeters were used as a check on precipitation data from the weighing lysimeter mass changes, but weighing lysimeter data were reported because the weighing lysimeters have a much larger effective orifice (~9 m^2^ compared with ~0.03 m^2^ for the rain gauge), and the lysimeter effective orifice is practically at ground surface so that wind has negligible effect on catch, unlike the case for the rain gauges at the weather station and weighing lysimeters. The weighing lysimeters typically report larger amounts of precipitation from large, quick storms than do the rain gauges, and they also typically are sensitive to small precipitation events that are not recorded by the tipping bucket rain gauges on the research station. Because convective thunderstorms common to the region have rain shafts that deliver precipitation to relatively small areas, precipitation can vary spatially. Therefore, precipitation is reported separately for each of the four lysimeters when available and a mean value is reported as well. Rainfall at Bushland comes primarily from these convective thunderstorms, some of which occur at night (see Fig. 3), and most of which are of short duration. In general, it is not possible to separate ET from rainfall. There are, however, mitigating factors that lessen the possible error in ET. For one, ET at night is often negligible, so not accounting for it during nighttime rainfall events is of little consequence. For another, humidity during extended rainfall events is typically near 100%, limiting transpiration and evaporation fluxes due to lack of driving gradient for these fluxes. Models and manufacturers of weather station sensors changed over the years as documented in the Excel spreadsheets for each year. Calibrated sensors were always used and in most cases sensors were replicated to provide data for quality control and gap filling^2^. Quality control and data gap filling were practiced as reported by Evett *et al*.^12^, and data are reported on a 15-minute basis for each year in the datasets in “Standard Quality Controlled Research Weather Data – USDA-ARS, Bushland, Texas”^11^.

## Data Records

All data records are stored on the USDA ARS National Agricultural Library Ag Data Commons (https://agdatacommons.nal.usda.gov/) in four dataset collections, one for each species studied. One is named, “The Bushland, Texas Alfalfa Datasets” (10.15482/USDA.ADC/1526356)^14^; one is named “The Bushland, Texas Soybean Datasets” (10.15482/USDA.ADC/1528779)^16^; one is named “The Bushland, Texas Sunflower Datasets (10.15482/USDA.ADC/1528066)^17^; and one is named the “Bushland, Texas Winter Wheat Datasets” (10.15482/USDA.ADC/1527912)^18^. For each species studied, there are six datasets, each containing one or more files:

1. Agronomic Calendars
2. Growth and Yield Data
3. Weighing Lysimeter Data
4. Evapotranspiration, Irrigation, Dew/frost - Water Balance
5. Standard Quality Controlled Research Weather Data – USDA-ARS, Bushland, Texas^11^
6. Soil Water Content Data for The Bushland, Texas Large Weighing Lysimeter Experiments^10^

Each dataset and the files in it are described in the following.

### Agronomic calendars

For the alfalfa crops there is one crop calendar spreadsheet for all years, including the fall of 1995, which is a resource available as file “1995–1999 Alfalfa Calendar.xlsx”, “Agronomic Calendar for the Bushland, Texas Alfalfa Datasets”. For the soybean, sunflower, and winter wheat crops, there is a crop calendar for each year for the two east lysimeters (NE and SE), when a crop was grown there, and another calendar for the two west lysimeters (NW and SW), when a crop was grown there. The agronomic calendar for each season lists by date the agronomic operations on the Bushland, TX, large weighing lysimeters and surrounding fields, including tillage, planting, fertilization, pesticide application, furrow diking, irrigations, etc., and also sensor installation, sensor reading that might disturb lysimeter operation (neutron probe readings), maintenance operations such as emptying drainage tanks, adjusting lysimeter scale counterweights, electronic and electrical maintenance, etc. Amounts and kinds of fertilizer and pesticide applications are given with proper chemical names and SI units. Calendars are in Excel files consisting of two tabs, the first a data dictionary, and the second containing the calendar.

Agronomic Calendars for the Bushland, Texas Soybean Datasets ^20^

Resources in this dataset:

- Resource Title: 1995 Bushland, TX, west soybean agronomic calendar. File Name: 1995 West Soybean Calendar(FINAL).xlsx.
- Resource Title: 2003 Bushland, TX, east soybean agronomic calendar. File Name: 2003 East Soybean Calendar (FINAL).xlsx.
- Resource Title: 2004 Bushland, TX, east soybean agronomic calendar. File Name: 2004 East Soybean Calendar (FINAL).xlsx.
- Resource Title: 2010 Bushland, TX, west soybean agronomic calendar. File Name: 2010 West Soybean Calendar(FINAL).xlsx.
- Resource Title: 2019 Bushland, TX, east soybean agronomic calendar. File Name: 2019 East Soybean Calendar(FINAL).xlsx.
- Resource Title: 2019 Bushland, TX, west soybean agronomic calendar. File Name: 2019 West Soybean Calendar(FINAL).xlsx.

Agronomic Calendars for the Bushland, Texas Sunflower Datasets ^21^

Resources in this dataset:

- Resource Title: 2009 Bushland, TX, east sunflower agronomic calendar. File Name: 2009 East Sunflower Calendar.xlsx.
- Resource Title: 2011 Bushland, TX, east sunflower agronomic calendar. File Name: 2011 East Sunflower Calendar.xlsx.

Agronomic Calendars for the Bushland, Texas Winter Wheat Datasets ^22^

Resources in this dataset:

- Resource Title: 1989–1990 Bushland, TX, west winter wheat agronomic calendar. File Name: 1989–1990 West Wheat Calendar.xlsx.
- Resource Title: 1991–1992 Bushland, TX, west winter wheat agronomic calendar. File Name: 1991–1992 East Wheat Calendar.xlsx.
- Resource Title: 1998 Bushland, TX, east alfalfa growth and yield data. File Name: 1998_alfalfa_plant_growth_&_yield.xlsx.

### Growth and yield data

Growth and yield data are contained in Excel files containing several tabs that list by date, field, and replicate number: crop growth [plant height, row width (for row crops), leaf area index, biomass (undried and dried), growth stage, etc.]; head or pod mass if present; population density; machine yield by field location; and hand (manual) harvest data per replicated plot (total biomass, dry yield, yield at standard moisture content). There was one growth and yield file for each year for the east (NE and SE) fields combined when a crop was grown on the east fields and one growth and yield file for the west (NW and SW) fields combined when a crop was grown on the west fields. Protocols for plant measurements and growth staging changed over the years, requiring different data dictionaries for different growing seasons. Protocols are explained in data dictionary tabs that precede each data tab. Data dictionaries explain the header for each column of data in the like-named data tab. Explanations include units of measure, plot size or row length sampled, and any pertinent methodological information. Data are from replicate (n = 3 or greater) samples in the field and non-destructive (except for final harvest) measurements on the weighing lysimeters. In most cases yield data are available from both manual sampling on replicate plots in each field and from machine harvest. The data dictionary and data tabs are preceded by an introduction tab that explains the other tab names and contents, team members, pertinent references, and conventions. Data are intermittent, meaning that if measurements were not taken on a date, then there is no date or data entry in the file.

Growth and Yield Data for the Bushland, Texas Alfalfa Datasets

(10.15482/USDA.ADC/1526355)^27^

Resources in this dataset:

- Resource Title: 1996 Bushland, TX, east alfalfa growth and yield data. File Name: 1996_alfalfa_plant_growth_&_yield.xlsx.
- Resource Title: 1997 Bushland, TX, east alfalfa growth and yield data. File Name: 1997_alfalfa_plant_growth_&_yield.xlsx.
- Resource Title: 1998 Bushland, TX, east alfalfa growth and yield data. File Name: 1998_alfalfa_plant_growth_&_yield.xlsx.
- Resource Title: 1999 Bushland, TX, east alfalfa growth and yield data. File Name: 1999_alfalfa_plant_growth_&_yield.xlsx.

Growth and Yield Data for the Bushland, Texas Soybean Datasets

(10.15482/USDA.ADC/1528670)^28^

Resources in this dataset:

- Resource Title: 1995 Bushland, TX, west soybean growth and yield data. File Name: 1995 West Soybean_Growth_and_Yield-V2.xlsx.
- Resource Title: 2003 Bushland, TX, east soybean growth and yield data. File Name: 2003 East Soybean_Growth_and_Yield-V2.xlsx.
- Resource Title: 2004 Bushland, TX, east soybean growth and yield data. File Name: 2004 East Soybean_Growth_and_Yield-V2.xlsx.
- Resource Title: 2010 Bushland, TX, west soybean growth and yield data. File Name: 2010 West Soybean_Growth_and_Yield-V2.xlsx.
- Resource Title: 2019 Bushland, TX, east soybean growth and yield data. File Name: 2019 East Soybean_Growth_and_Yield-V2.xlsx.
- Resource Title: 2019 Bushland, TX, west soybean growth and yield data. File Name: 2019 West Soybean_Growth_and_Yield-V2.xlsx.

Growth and Yield Data for the Bushland, Texas Sunflower Datasets

(10.15482/USDA.ADC/1528072)^29^

Resources in this dataset:

- Resource Title: 2009 Bushland, TX, east sunflower growth and yield data. File Name: 2009_East_Sunflower_Growth_and_Yield.xlsx.
- Resource Title: 2011 Bushland, TX, east sunflower growth and yield data. File Name: 2011_East_Sunflower_Growth_and_Yield.xlsx.

Growth and Yield Data for the Bushland, Texas Winter Wheat Datasets

(10.15482/USDA.ADC/1527918)^30^

Resources in this dataset:

- Resource Title: 1989–1990 Bushland, TX, west winter wheat growth and yield data. File Name: 1989–1990_West_Wheat_Growth_and_Yield.xlsx.
- Resource Title: 1991–1992 Bushland, TX, east winter wheat growth and yield data. File Name: 1991–1992_East_Wheat_Growth_and_Yield.xlsx.
- Resource Title: 1992–1993 Bushland, TX, west winter wheat growth and yield data. File Name: 1992–1993_W_Wheat_Growth_and_Yield.xlsx.

### Weighing lysimeter data

This dataset contains lysimeter soil water storage and drainage data, which are converted to equivalent depth of water per unit area from lysimeter mass data, as well as data from in-soil and above-soil sensors. Each lysimeter was equipped with a suite of instruments to sense lysimeter mass, wind speed, air temperature and relative humidity, components of the radiation balance (e.g., net radiation, incoming and reflected shortwave, photosynthetically active radiation (PAR) in some years, incoming and upwelling longwave, thermal infrared emitted by the plant/soil surface), soil heat flux, soil temperature, and soil volumetric water content at certain depths (in later years). Data are on 5-minute, 15-minute, or daily basis for all days of the year with missing data indicated by #N/A. Although a quality control process was used, the evapotranspiration (ET) data in this dataset are considered raw data. Advanced algorithms for detection of precipitation, dew and frost were applied in a separate process to determine higher quality ET values that are reported in files in datasets titled in part “Evapotranspiration and Water Balance Data” (see following section). Those files have “water-balance” in their names. Not all properties were always sensed in any one year; and instruments used changed from season to season, which are reasons that subsidiary datasets and data dictionaries for each season are required.

There is one file for each year for the east (NE and SE) lysimeters combined when a crop was grown there and one file for each year for the west (NW and SW) lysimeters when a crop was grown on the west fields and lysimeters. Data are in Excel files consisting of an introductory tab followed by data dictionary and data tabs. The data tab for 5-minute data contains only data that were reported on a 5-minute mean basis, typically lysimeter relative water storage, standard deviation of same, rain gauge data, again in mm equivalent depth of water. In the 5-minute data tab, there are columns for data flags for water storage values for each lysimeter, and the meaning of the flags is given in the introductory tab. These flags typically indicate the occurrences of precipitation, dew or frost accumulation, lysimeter counterweight adjustment, lysimeter maintenance, drainage tank emptying, and other occurrences that would have influenced the lysimeter mass other than the evapotranspiration process itself. A tab for 15-minute mean data values contains data for lysimeter relative water storage and all other data from sensors in and above the lysimeter. A daily tab contains daily mean or total data for the same sensors and the corresponding data dictionary explains the units of measure, instruments used, and methods used. The daily data tab is followed by several tabs that are used for data visualization and do not contain data.

Weighing Lysimeter Data for The Bushland, Texas Alfalfa Datasets

(10.15482/USDA.ADC/1526357)^31^

Resources in this dataset:

- Resource Title: 1996 Bushland, TX, East Alfalfa Weighing Lysimeter and Microclimate Data. File Name: 1996_Alfalfa_E_Lys_ClimDat.xlsx.
- Resource Title: 1997 Bushland, TX, East Alfalfa Weighing Lysimeter and Microclimate Data. File Name: 1997_Alfalfa_E_Lys_ClimDat.xlsx.
- Resource Title: 1998 Bushland, TX, East Alfalfa Weighing Lysimeter and Microclimate Data. File Name: 1998_Alfalfa_E_Lys_ClimDat.xlsx.
- Resource Title: 1999 Bushland, TX, East Alfalfa Weighing Lysimeter and Microclimate Data. File Name: 1999_Alfalfa_E_Lys_ClimDat.xlsx.

Weighing Lysimeter Data for The Bushland, Texas Soybean Datasets

(10.15482/USDA.ADC/1528684)^32^

Resources in this dataset:

- Resource Title: 1995 Bushland, TX, West Soybean Weighing Lysimeter and Microclimate Data. File Name: 1995_Soybean_W_Lys_ClimDat(FINAL).xlsx.
- Resource Title: 2003 Bushland, TX, East Soybean Weighing Lysimeter and Microclimate Data. File Name: 2003_Soybean E_Lys_ClimDat(FINAL).xlsx.
- Resource Title: 2004 Bushland, TX, East Soybean Weighing Lysimeter and Microclimate Data. File Name: 2004_Soybean E_Lys_ClimDat(FINAL).xlsx.
- Resource Title: 2010 Bushland, TX, West Soybean Weighing Lysimeter and Microclimate Data. File Name: 2010_Soybean_W_Lys_ClimDat(FINAL).xlsx.
- Resource Title: 2019 Bushland, TX, East Soybean Weighing Lysimeter and Microclimate Data. File Name: 2019_Soybean_E_Lys_ClimDat(FINAL).xlsx.
- Resource Title: 2019 Bushland, TX, West Soybean Weighing Lysimeter and Microclimate Data. File Name: 2019_Soybean_W_Lys_ClimDat(FINAL).xlsx.

Weighing Lysimeter Data for The Bushland, Texas Sunflower Datasets

(10.15482/USDA.ADC/1528074)^33^

Resources in this dataset:

- Resource Title: 2009 Bushland, TX, East Sunflower Weighing Lysimeter and Microclimate Data. File Name: 2009_East_Sunflower_Lys_ClimDat.xlsx.
- Resource Title: 2011 Bushland, TX, East Sunflower Weighing Lysimeter and Microclimate Data. File Name: 2011_East_Sunflower_Lys_ClimDat.xlsx.

Weighing Lysimeter Data for The Bushland, Texas Winter Wheat Datasets

(10.15482/USDA.ADC/1527916)^34^

Resources in this dataset:

- Resource Title: 1989 Bushland, TX, West Winter Wheat Weighing Lysimeter and Microclimate Data. File Name: 1989_Wheat W_Lys_ClimDat.xlsx.
- Resource Title: 1990 Bushland, TX, West Winter Wheat Weighing Lysimeter and Microclimate Data. File Name: 1990_Wheat W_Lys_ClimDat.xlsx.
- Resource Title: 1991 Bushland, TX, East Winter Wheat Weighing Lysimeter and Microclimate Data. File Name: 1991_Wheat_E_Lys_ClimDat.xlsx.
- Resource Title: 1992 Bushland, TX, East Winter Wheat Weighing Lysimeter and Microclimate Data. File Name: 1992_Wheat_E_Lys_ClimDat.xlsx.
- Resource Title: 1992 Bushland, TX, West Winter Wheat Weighing Lysimeter and Microclimate Data. File Name: 1992_Wheat_W_Lys_ClimDat.xlsx.
- Resource Title: 1993 Bushland, TX, West Winter Wheat Weighing Lysimeter and Microclimate Data. File Name: 1993_Wheat_W_Lys_ClimDat.xlsx.

### Evapotranspiration, Irrigation, Dew/frost - Water Balance Data

Values in the water balance datasets are the result of a rigorous quality control process involving algorithms for detecting dew/frost accumulations, and precipitation (rain and snow)^24,25^. The ET, precipitation, and irrigation data in this dataset should be considered to be the most accurate values offered in these datasets. Even though ET data are also presented in the “lysimeter” datasets, the values herein are the result of a more rigorous quality control process. The water balance data consist of 15-minute and daily amounts of evapotranspiration (ET), dew/frost accumulation, precipitation (rain/snow), irrigation, scale counterweight adjustment, and emptying of drainage tanks, all in mm equivalent depth of water. There are separate data tabs for the daily and 15-minute data, and a third data tab that gives irrigation amounts and irrigation application method for each date on which irrigation was applied. A data dictionary for each data tab explains units of measurement, methods of measurement, methods of irrigation, or other information pertinent to each data column header. Changes in lysimeter mass due to emptying of drainage tanks, counterweight adjustment, maintenance activity, and harvest are accounted for such that ET values are minimally affected. Dew and frost accumulation varies from year to year and seasonally within a year, and it is affected by lysimeter surface condition [bare soil, tillage condition, residue amount and orientation (flat or standing), etc.]. Particularly during winter and depending on humidity and cloud cover, dew and frost accumulation sometimes accounts for an appreciable percentage of total daily ET. An introductory tab explains the other tabs, gives lists of authors and references, and explains conventions and symbols used. It also lists instruments and data recorders used, and longitude and latitude of lysimeter assets, weather station, and field corners. There is one file for each year for the east (NE and SE) fields combined.

Evapotranspiration, Irrigation, Dew/frost - Water Balance Data for The Bushland, Texas Alfalfa Datasets

(10.15482/USDA.ADC/1526370)^35^

Resources in this dataset:

- Resource Title: 1996 Bushland, TX. East Alfalfa Evapotranspiration, Irrigation, and Water Balance Data. File Name: 1996_alfalfa_water_balance.xlsx
- Resource Title: 1997 Bushland, TX. East Alfalfa Evapotranspiration, Irrigation, and Water Balance Data. File Name: 1997_alfalfa_water_balance.xlsx
- Resource Title: 1998 Bushland, TX. East Alfalfa Evapotranspiration, Irrigation, and Water Balance Data. File Name: 1998_alfalfa_water_balance.xlsx
- Resource Title: 1999 Bushland, TX. East Alfalfa Evapotranspiration, Irrigation, and Water Balance Data. File Name: 1999_alfalfa_water_balance.xlsx

Evapotranspiration, Irrigation, Dew/frost - Water Balance Data for The Bushland, Texas Soybean Datasets

(10.15482/USDA.ADC/1528713)^26^

Resources in this dataset:

- Resource Title: 1995 Bushland, TX, West Soybean Evapotranspiration, Irrigation, and Water Balance Data. File Name: 1995_W_Soybean_water_balance.xlsx
- Resource Title: 2003 Bushland, TX, East Soybean Evapotranspiration, Irrigation, and Water Balance Data. File Name: 2003_E_Soybean_water_balance.xlsx
- Resource Title: 2004 Bushland, TX, East Soybean Evapotranspiration, Irrigation, and Water Balance Data. File Name: 2004_E_Soybean_water_balance.xlsx
- Resource Title: 2010 Bushland, TX, West Soybean Evapotranspiration, Irrigation, and Water Balance Data. File Name: 2010_W_Soybean_water_balance.xlsx
- Resource Title: 2019 Bushland, TX, East Soybean Evapotranspiration, Irrigation, and Water Balance Data. File Name: 2019_E_Soybean_water_balance.xlsx
- Resource Title: 2019 Bushland, TX, West Soybean Evapotranspiration, Irrigation, and Water Balance Data. File Name: 2019_W_Soybean_water_balance.xlsx

Evapotranspiration, Irrigation, Dew/frost - Water Balance Data for The Bushland, Texas Sunflower Datasets

(10.15482/USDA.ADC/1528081)^36^

Resources in this dataset:

- Resource Title: 2009 Bushland, TX. East Sunflower Evapotranspiration, Irrigation, and Water Balance Data. File Name: 2009_E_Sunflower_water_balance.xlsx
- Resource Title: 2011 Bushland, TX. East Sunflower Evapotranspiration, Irrigation, and Water Balance Data. File Name: 2011_E_Sunflower_water_balance.xlsx

Evapotranspiration, Irrigation, Dew/frost - Water Balance Data for The Bushland, Texas Winter Wheat Datasets

(10.15482/USDA.ADC/1527917)^37^

Resources in this dataset:

- Resource Title: 1989 Bushland, TX. West Winter Wheat Evapotranspiration, Irrigation, and Water Balance Data. File Name: 1989_W_Wheat_water_balance.xlsx
- Resource Title: 1990 Bushland, TX. West Winter Wheat Evapotranspiration, Irrigation, and Water Balance Data. File Name: 1990_W_Wheat_water_balance.xlsx
- Resource Title: 1991 Bushland, TX. East Winter Wheat Evapotranspiration, Irrigation, and Water Balance Data. File Name: 1991_E_Wheat_water_balance.xlsx
- Resource Title: 1992 Bushland, TX. East Winter Wheat Evapotranspiration, Irrigation, and Water Balance Data. File Name: 1992_E_Wheat_water_balance.xlsx
- Resource Title: 1992 Bushland, TX. West Winter Wheat Evapotranspiration, Irrigation, and Water Balance Data. File Name: 1992_W_Wheat_water_balance.xlsx
- Resource Title: 1993 Bushland, TX. West Winter Wheat Evapotranspiration, Irrigation, and Water Balance Data. File Name: 1993_W_Wheat_water_balance.xlsx

### Quality controlled weather data

Quality-controlled^12^ weather data for all days in each year, either as 15-minute means or totals, are available in these datasets from the USDA-ARS Conservation and Production Laboratory (CPRL), Soil and Water Management Research Unit (SWMRU) research weather station, Bushland, Texas (Lat. 35.186714°, Long. −102.094189°, elevation 1170 m above MSL). The data are from sensors placed at 2-m height over a level, grass surface mowed to not exceed 0.12-m height and irrigated and fertilized to maintain reference conditions^15,38^. Irrigation was by surface flood in 1989 through 1994, and by subsurface drip irrigation after 1994. The primary paper^12^ describes details of the sensors used and methods of testing, calibration, inter-comparison, and use. Sensors were replicated and intercompared between replicates and with data from nearby weather stations, which were sometimes used for gap filling. Data from a duplicate sensor were used to fill gaps in data from the primary sensor using appropriate regression relationships. Gap filling was also accomplished using sensors deployed at one of the four large weighing lysimeters immediately west of the weather station or using sensors at other nearby stations when reliable regression relationships could be developed. An important secondary station is the CPRL National Weather Service station. The weather data include air temperature (°C) and relative humidity (%)^39^, wind speed (m s^−1^), solar irradiance (W m^−2^), barometric pressure (kPa), and precipitation (rain and snow in mm). Because the large (3 m by 3 m surface area) weighing lysimeters are better rain gages than are tipping bucket gages, the 15-minute precipitation data are derived for each lysimeter from changes in lysimeter mass.

There is a separate Excel file containing data for each year. An introductory tab explains what are in the other tabs, lists authors and references, explains conventions and symbols, lists instrumentation used, including for the CPRL National Weather Service station, and gives the latitude and longitude of stations. The “15-minute weather” tab gives solar irradiance, air temperature and humidity, wind speed, air pressure, the precipitation at each of the four lysimeters and a mean precipitation value. The “daily precip. data” tab gives precipitation from each lysimeter and the mean precipitation for every day of the year. A data dictionary precedes each data tab. Additional tabs are included for data visualization, including a graphical representation of missing data, and a graphical representation of the weather data in 5-day segments as chosen by the user.

Standard Quality Controlled Research Weather Data – USDA-ARS, Bushland, Texas

(10.15482/USDA.ADC/1526433)^11^

Resources in this dataset (only files for the years in which alfalfa, soybean, and winter wheat were grown are listed):

- Resource Title: 1989 Bushland, TX, standard 15-minute weather data. File Name: 1989_15-min_weather_SWMRU_CPRL.xlsx.
- Resource Title: 1990 Bushland, TX, standard 15-minute weather data. File Name: 1990_15-min_weather_SWMRU_CPRL.xlsx.
- Resource Title: 1991 Bushland, TX, standard 15-minute weather data. File Name: 1991_15-min_weather_SWMRU_CPRL.xlsx.
- Resource Title: 1992 Bushland, TX, standard 15-minute weather data. File Name: 1992_15-min_weather_SWMRU_CPRL.xlsx.
- Resource Title: 1993 Bushland, TX, standard 15-minute weather data. File Name: 1993_15-min_weather_SWMRU_CPRL.xlsx.
- Resource Title: 1995 Bushland, TX, standard 15-minute weather data. File Name: 1995_15-min_weather_SWMRU_CPRL.xlsx.
- Resource Title: 1996 Bushland, TX, standard 15-minute weather data. File Name: 1996_15-min_weather_SWMRU_CPRL.xlsx.
- Resource Title: 1997 Bushland, TX, standard 15-minute weather data. File Name: 1997_15-min_weather_SWMRU_CPRL.xlsx.
- Resource Title: 1998 Bushland, TX, standard 15-minute weather data. File Name: 1998_15-min_weather_SWMRU_CPRL.xlsx.
- Resource Title: 1999 Bushland, TX, standard 15-minute weather data. File Name: 1999_15-min_weather_SWMRU_CPRL.xlsx.
- Resource Title: 2003 Bushland, TX, standard 15-minute weather data. File Name: 2003_15-min_weather_SWMRU_CPRL.xlsx.
- Resource Title: 2004 Bushland, TX, standard 15-minute weather data. File Name: 2004_15-min_weather_SWMRU_CPRL.xlsx.
- Resource Title: 2009 Bushland, TX, standard 15-minute weather data. File Name: 2009_15-min_weather_SWMRU_CPRL.xlsx.
- Resource Title: 2010 Bushland, TX, standard 15-minute weather data. File Name: 2010_15-min_weather_SWMRU_CPRL.xlsx.
- Resource Title: 2011 Bushland, TX, standard 15-minute weather data. File Name: 2011_15-min_weather_SWMRU_CPRL.xlsx.
- Resource Title: 2019 Bushland, TX, standard 15-minute weather data. File Name: 2019_15-min_weather_SWMRU_CPRL.xlsx.

### Soil water content data

For each year, a dataset in an Excel spreadsheet contains soil water content data developed from neutron probe readings taken in access tubes in each of the four large, precision weighing lysimeters and in the fields surrounding each lysimeter. Readings were taken periodically with a field-calibrated^13,40,41^ neutron probe at depths from 0.10 m to 2.30 m (maximum of 1.90 m depth in the lysimeters) in 0.20-m depth increments. Field calibrations in the Pullman soil series were done every few years. Calibrations typically produced a regression equation with RMSE ≤ 0.01 m^3^ m^3^.^42^ Data were used to guide irrigation scheduling to achieve full or deficit irrigation as required by the experimental design. Periods between readings were typically one to two weeks, sometimes longer according to experimental design, need for data, and weather that could prevent entry into the field. Data may be used to calculate the soil profile water content in mm of water from the surface to the maximum depth of reading. Profile water content differences between reading times in the same access tube are considered the change in soil water storage (∆*S*, mm) during the period in question and may be used to compute evapotranspiration (ET) using Eq. 1 with accuracy comparable to that for the weighing lysimeter for periods of a week or longer^43^.

Spreadsheets have several tabs, not all of which are data tabs; each data tab is preceded by a data dictionary tab explaining the measurement units and method for each column of data. The first tab is an introductory tab explaining the other tabs, listing the authors, and key references and listing conventions and explaining symbols. The first data tab gives profile water contents in the top 1.5 m of soil and also in the profile to 2.4-m depth. Profile water contents are given as mean values for each lysimeter (mean of readings in two access tubes) and field (from four to eight access tubes depending on the year, more for later years). Profile water contents are also given as mean values for every two access tubes in the field. There are two files for each year, one for east (NE and SE) fields, and one for west (NW and SW) fields. Depthwise soil volumetric water content (VWC) values are given in a tab for each day of reading, each depth, and each access tube number. The profile water content data are synthesized from these depthwise data. A “Tube Map” tab gives the location for each access tube by its number. Locations are relative to the fields and lysimeters and in later years are given in latitude and longitude. A “Graph” tab allows visualization of the data with one graph for each access tube illustrating the data for all dates of measurement.

Soil Water Content Data for The Bushland, Texas Large Weighing Lysimeter Experiments

(10.15482/USDA.ADC/1526332)^10^

Resources in this dataset (only files for the season years in which alfalfa, soybean, and winter wheat were grown are listed):

- Resource Title: 1989–90 Bushland, TX, west winter wheat volumetric soil water content data. File Name: 1989–90_West_Winter-Wheat_Soil-water.xlsx
- Resource Title: 1991–92 Bushland, TX, east winter wheat volumetric soil water content data. File Name: 1991–92_East_Winter-Wheat_Soil-water.xlsx
- Resource Title: 1992–93 Bushland, TX, west winter wheat volumetric soil water content data. File Name: 1992–93_West_Winter-Wheat_Soil-water.xlsx
- Resource Title: 1995 Bushland, TX, west soybean volumetric soil water content data. File Name: 1995_West_Soybean_Soil-water_0.xlsx
- Resource Title: 1996 Bushland, TX, east alfalfa volumetric soil water content data. File Name: 1996_East_Alfalfa_Soil-water (1).xlsx
- Resource Title: 1997 Bushland, TX, east alfalfa volumetric soil water content data. File Name: 1997_East_Alfalfa_Soil-water_0.xlsx
- Resource Title: 1998 Bushland, TX, east alfalfa volumetric soil water content data. File Name: 1998_East_Alfalfa_Soil-water_0.xlsx
- Resource Title: 1999 Bushland, TX, east alfalfa volumetric soil water content data. File Name: 1999_East_Alfalfa_Soil-water_0.xlsx
- Resource Title: 2003 Bushland, TX, east soybean volumetric soil water content data. File Name: 2003_East_Soybean_Soil-water.xlsx
- Resource Title: 2004 Bushland, TX, east soybean volumetric soil water content data. File Name: 2004_East_Soybean_Soil-water.xlsx
- Resource Title: 2009 Bushland, TX, east sunflower volumetric soil water content data. File Name: 2009_East_Sunflower_Soil-water.xlsx
- Resource Title: 2010 Bushland, TX, west soybean volumetric soil water content data. File Name: 2010_West_Soybean_Soil-water.xlsx
- Resource Title: 2011 Bushland, TX, east sunflower volumetric soil water content data. File Name: 2011_East_Sunflower_Soil-water.xlsx
- Resource Title: 2019 Bushland, TX, east soybean volumetric soil water content data. File Name: 2010_East_Soybean_Soil-water.xlsx
- Resource Title: 2019 Bushland, TX, west soybean volumetric soil water content data. File Name: 2019_West_Soybean_Soil-water.xlsx

## Technical Validation

Several studies have aimed to improve and maintain quality of the Bushland datasets. Weighing lysimeter accuracy ranging from 0.01- to 0.05-mm equivalent water depth has been demonstrated in published calibration results^44,45^. Determination of soil water content depended on methods of use and calibration for neutron probes developed at Bushland and widely reported in journal articles^13,39,46^ and book chapters^41,47–49^. The use of the neutron probe to accurately determine the profile water content from the soil surface to various depths and the equivalency of weighing lysimeter ET to that calculated from neutron probe based soil water balance for periods of a week or longer was demonstrated^43,44^. There is a question of how representative weighing lysimeter ET is of field ET because weighing lysimeters represent a relatively small surface area compared to the much larger field. It was demonstrated that a network of neutron probe access tubes in the field around a lysimeter could be used to show that the lysimeter ET was representative of the field ET when crop cover on the lysimeter was essentially the same as that in the field^44^. Other than changes due to evapotranspiration (ET), lysimeter apparent relative water storage will change due to irrigation, precipitation, dew or frost accumulation, scale counterweight adjustments, work on the lysimeter such as neutron probe readings, and other events. Correcting for these non-ET apparent storage changes allows ET to be calculated from the corrected lysimeter storage. As partially illustrated in Fig. 3, methods for detecting these events and making the corrections were developed and documented^12,24,25^.

**Fig. 4:**
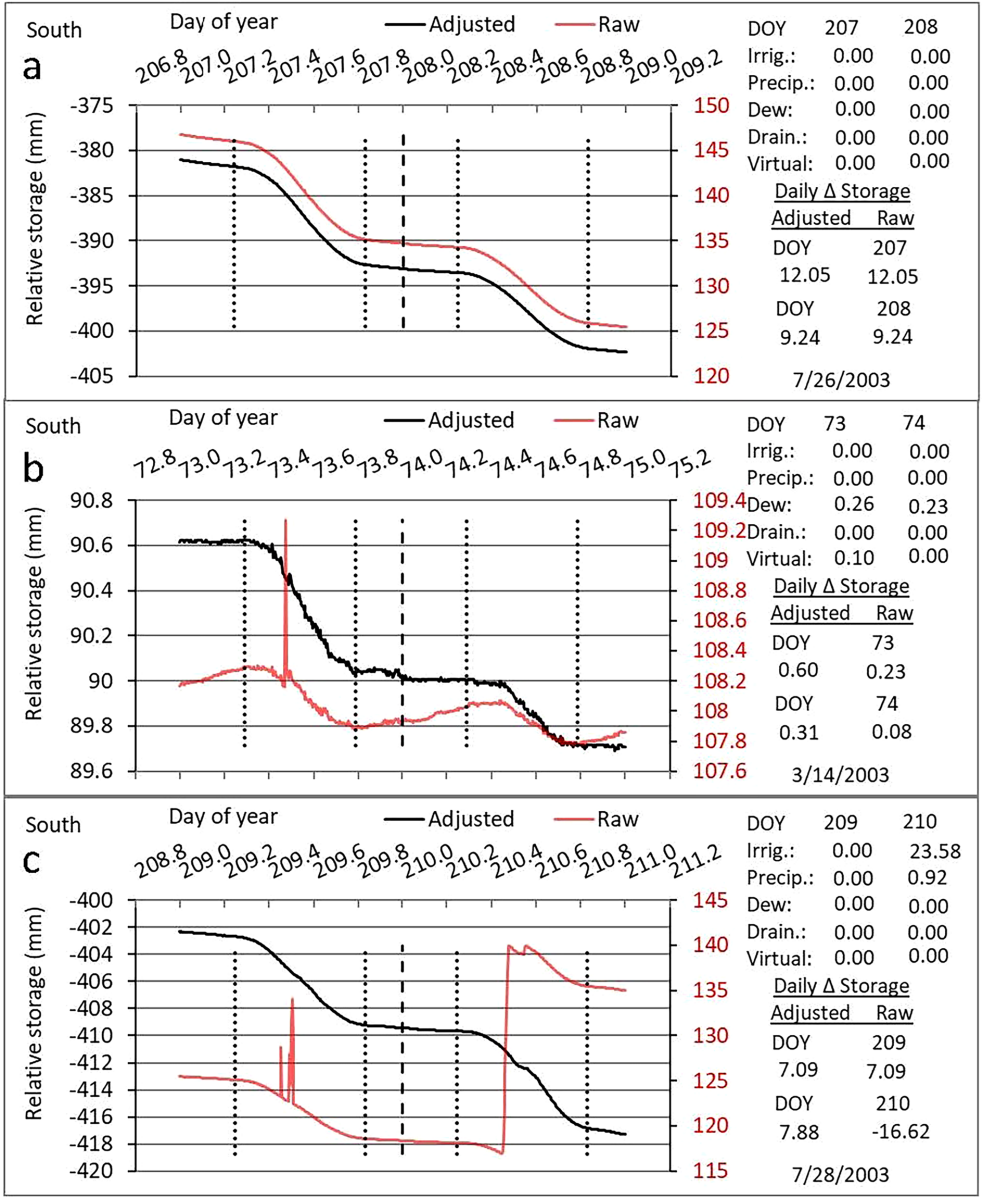
Illustrations of raw lysimeter data (red) in 2003 showing the change of relative storage (mass) over time due to various events, and adjusted data (black) after the events are taken into account such that relative storage change reflects only evapotranspiration (ET). Vertical dotted lines indicate sunrise and sunset; vertical dashed line indicates midnight. (**a**) Change in relative storage over time on two days of year (DOY) when no events other than ET caused changes in lysimeter mass. Nighttime ET was evident. (**b**) Illustration of raw lysimeter data with a spike on DOY 73 when work on the lysimeter caused the spike and a resulting small increase in lysimeter mass (relative storage). Also shown are nighttime increases in relative storage due to dewfall. The adjusted relative storage accounted for both sources of mass increase. (**c**) Change in relative storage over time when work on the lysimeter caused two spikes on DOY 209, and an irrigation followed by a small precipitation event caused increases in raw relative storage on DOY 210. The adjusted relative storage accounted for all these events.

A year-long sequence of 5-minute lysimeter storage data is contained in an example spreadsheet^20^ available on the Ag Data Commons to demonstrate the algorithms and calculations made to correct all the events illustrated in Figs. 3 & 4. These and other corrections are discussed in the 10 papers cited in the spreadsheet posting. In addition to or in lieu of the data processing steps described herein, several lysimeter data processing schemes have been published, all of which included some form of quality control and event flagging steps. Five steps were included in one scheme: a manual filter (e.g., removal of I, P, or maintenance events), threshold filter (e.g., removal of spikes), median filter (single outliers or smaller spikes), smoothing (moving average with 15-min window), and oscillation threshold filter (further smoothing), where interpolation was used to fill gaps following the manual filter and threshold filter steps^50^. Another scheme^51^ used Savitsky-Golay^52^ smoothing of lysimeter storage and its first derivative, which is ET, after a manual filtering step. Some schemes assumed that P and ET do not occur simultaneously, so that gap filling of ET was not required during P events^53–56^. These latter schemes were developed for non-irrigated bare soil or natural vegetated surfaces. Our methods may be more time consuming because they require user intervention to apply flags and correct automatically assigned flags where necessary. However, we consider our methods to be superior because they do not lose detailed information through application of smoothing.

At two or more weather stations, multiple redundant measurements were made of important properties (air temperature and RH, wind speed and solar irradiance) for intercomparison and replacement of faulty or missing data. Quality assurance and control were also achieved through calibration of relative humidity sensors (using salt solutions and a calibration chamber)^39,57^, soil heat flux plates^58^, radiation sensors (routinely sent to manufacturers for calibration), anemometers (in a test stand), and tipping-bucket rain gauges (comparison to lysimeter mass change and to U.S. Weather Bureau standard rain gauges). Solar irradiance is compared to theoretical clear-sky radiation both to gauge sensor performance and to check on data time stamping^12,45^.

Replicate (n = 3 or more) plots randomly assigned in each field were used for plant growth and yield sampling and observation. Individual values were reported so that mean and standard deviation values could be computed and statistical analysis applied. Alfalfa plant growth stages were observed on the replicate plots and recorded according to the Kalu and Fick growth scale^59^. Soybean plant growth stages were observed and recording according to Berglund *et al*.^60^. Sunflower growth stages were assessed and recorded as vegetative or growth stages^61^. Winter wheat growth stages were observed on the replicate plots and recorded according to the Feekes growth scale^62^. Harvest index was calculated from grain yield and above ground biomass values, in part as a check against values reported in the literature to assess systematic errors^4^.

## Usage Notes

A number of papers document efforts using these datasets to intercompare, test and improve reference evapotranspiration models^15,63–66^, to develop crop coefficients for estimation of crop water use in combination with a reference evapotranspiration model^3–6,67^, and to intercompare, test and calibrate simulation models of crop growth, energy and water balance, water use (ET), and yield^8,68–70^. Soil property values, necessary for simulation modelling, are available as a resource (Bushland_TX_soil_properties.xlsx) in the alfalfa dataset collection^14^. Care was taken to ensure that lysimeter ET data were representative of the 4.4 ha fields within which each lysimeter was centered. Satellite data with 100-m or smaller pixels thus may be suitable for use with the lysimeter data in testing and calibration of models based on satellite data, and they have been so used. Nevertheless, the data pertain to the specific location, soil, climate, cultivar, and agronomic practices described in the data sets. Extrapolation to other climates, soils, cultivars, and practices should be done with care. Individual fields were square and somewhat larger than 210 m in width and length, so care should be used when combining satellite data with these data if satellite image pixels are large. Observations of air temperature and relative humidity, wind speed, and solar irradiance taken at the lysimeters should not be used as weather input for simulation models; rather, weather data observed under standard conditions at the research weather station should be used as input to simulation models.

## Data Availability

All data records are stored on the USDA ARS National Agricultural Library Ag Data Commons (https://agdatacommons.nal.usda.gov/) in four dataset collections, one for each species studied. For alfalfa (*Medicago sativa* L.), the collection is named, “The Bushland, Texas Alfalfa Datasets” (10.15482/USDA.ADC/1526356)^14^. For soybean (*Glycine max* (L.) Merr.), the collection is named “The Bushland, Texas Soybean Datasets” (10.15482/USDA.ADC/1528779)^16^. For sunflower (*Helianthus annuus* L.), the collectdion is named The Bushland, Texas Sunflower Datasets (10.15482/USDA.ADC/1528066)^17^. For winter wheat (*Triticum aestivum*, L.), the collection is named the “Bushland, Texas Winter Wheat Datasets” (10.15482/USDA.ADC/1527912)^18^. There are six datasets for each species studied, each containing one or more files: 1. Agronomic Calendars for alfalfa^14^, soybean^20^, sunflower^21^, and winter wheat^22^ 2. Growth and Yield Data for alfalfa^27^, soybean^28^, sunflower^29^, and winter wheat^30^ 3. Weighing Lysimeter Data for alfalfa^31^, soybean^32^, sunflower^33^, and winter wheat^34^ 4. Evapotranspiration, Irrigation, Dew/frost - Water Balance data for alfalfa^35^, soybean^26^, sunflower^36^, and winter wheat^37^ 5. Standard Quality Controlled Research Weather Data – USDA-ARS, Bushland, Texas^11^ 6. Soil Water Content Data for The Bushland, Texas Large Weighing Lysimeter Experiments^10^. Each dataset and the files in it are described in the preceding sections. Soil property values needed for simulation modelling are available as a resource (Bushland_TX_soil_properties.xlsx) in the alfalfa dataset collection^14^. The spreadsheet used to analyse weighing lysimeter water storage data and produce values of evapotranspiration, dew and frost accumulation, irrigation, and precipitation amounts is available on the USDA ARS NAL Ag Data Commons (10.15482/USDA.ADC/26898151)^71^.

## Code availability

A custom Excel spreadsheet was used to identify changes in apparent lysimeter water storage caused by other than evapotranspiration (ET) and to correct the lysimeter water storage so that it reflected only ET, enabling calculation of ET (see Figs. 3 & 4). The spreadsheet was essentially an embodiment of Eq. 2. The spreadsheet allowed separate tabulation of amounts of precipitation, irrigation, dew and frost accumulation, and drainage tank emptying. The spreadsheet is available on the USDA ARS NAL Ag Data Commons (10.15482/USDA.ADC/26898151)^71^.

## References

[1] Marek, T. H., Schneider, A. D., Howell, T. A. & Ebeling, L. L. Design and construction of large weighing monolithic lysimeters. *Trans. ASAE* **31**(2), 477–484, 10.13031/2013.30734 (1988).

[2] Evett, S. R. *et al*. The Bushland Weighing Lysimeters: A Quarter Century of Crop ET Investigations to Advance Sustainable Irrigation. *Trans. ASABE* **59**(1), 163–179, 10.13031/trans.59.11159 (2016).

[3] Evett, S. R., Marek, G. W., Colaizzi, P. D., Brauer, D. & Howell, T. A. Are Crop Coefficients for SDI Different from Those for Sprinkler Irrigation Application? *Trans. ASABE* **63**(5), 1233–1242, 10.13031/trans.13920 (2020).

[4] Howell, T. A. *et al*. Crop Coefficients Developed at Bushland, Texas for Corn, Wheat, Sorghum, Soybean, Cotton, and Alfalfa. *World Environmental and Water Resource Congress*10.1061/40856(200)291 (2006).

[5] Marek, T. H., Howell, T. A., Snyder, R. L., Porter, D. & Scherer, T. Crop coefficient development and application to an evapotranspiration network. Paper number IRR10-9786, *5th National Decennial Irrigation Conference Proceedings*, 5-8 December 2010, Phoenix Convention Center, Phoenix, Arizona USA. ASABE, St. Joseph, MI USA. 10.13031/2013.35815 (2010).

[6] Evett, S.R., T.A. Howell, A.D. Schneider and J.A. Tolk. Crop coefficient based evapotranspiration estimates compared with mechanistic model results. pp. 1585-1589. In W.H. Espey, Jr. and P. G. Comps (eds.) Vol. 2, *Water Resources Engineering*. *Am. Soc. Civil. Engr.*, New York, NY. (1995).

[7] Evett, S. R., Howell, T. A., Schneider, A. D., Copeland, K. S. & Dusek, D. A. Energy and water balance modeling of winter wheat. ASAE Paper No. 94-2022 (1994).

[8] Colaizzi, P. D., Evett, S. R., Howell, T. A. & Tolk, J. A. Lysimetric evaluation of single- and two-source energy balance models for alfalfa, grain sorghum, and cotton in the Southern High Plains. *Impacts of Global Climate Change*10.1061/40792(173)543 (2005).

[9] Kang, S., Payne, W. A., Evett, S. R., Robinson, C. A. & Stewart, B. A. Simulation of winter wheat evapotranspiration in Texas and Henan using three models of differing complexity. *Agricultural Water Management*, **96**(1), 167–178 10.1016/j.agwat.2008.07.006 (2009).

[10] Evett, S. R. *et al*. Soil Water Content Data for The Bushland, Texas Large Weighing Lysimeter Experiments. *Ag Data Commons*10.15482/USDA.ADC/1526332 (2022).

[11] Evett, S. R. Standard Quality Controlled Research Weather Data – USDA-ARS, Bushland, Texas. *Ag Data Commons*10.15482/USDA.ADC/1526433 (2022).

[12] Evett, S. R., Marek, G. W., Copeland, K. S. & Colaizzi, P. D. Quality Management for Research Weather Data: USDA-ARS, Bushland, TX. *Agrosyst. Geosci. Environ.* **1**(2018), 180036, 10.2134/age2018.09.0036 (2018).

[13] Evett, S. R., Marek, G. W., Colaizzi, P. D., Copeland, K. S. & Ruthardt, B. B. Methods for downhole soil water sensor calibration—Complications of bulk density and water content variations. *Vadose Zone J*. e20235, 10.1002/vzj2.20235 (2022).

[14] Evett, S. R. *et al*. The Bushland, Texas, Alfalfa Datasets. *USDA ARS NAL Ag Data Commons*10.15482/USDA.ADC/1526356 (2022).

[15] ASCE. The ASCE Standardized Reference Evapotranspiration Equation. Technical Committee on Standardization of Reference Evapotranspiration; Edited by Richard G. Allen, Ivan A. Walter, Ronald L. Elliott, Terry A. Howell, Daniel Itenfisu, Marvin E. Jensen, and Richard L. Snyder. ISBN (print): 9780784408056ISBN (PDF): 9780784475638, 10.1061/9780784408056 (2005).

[16] Evett, S. R. *et al*. The Bushland, Texas Soybean Datasets. *Ag Data Commons*10.15482/USDA.ADC/1528779 (2023).

[17] Evett, S. R. *et al*. The Bushland, Texas Sunflower Datasets. *Ag Data Commons*10.15482/USDA.ADC/1528066 (2022).

[18] Evett, S. R. *et al*. The Bushland, Texas Winter Wheat Datasets. *USDA ARS NAL Ag Data Commons*10.15482/USDA.ADC/1527912 (2022).

[19] Evett, S. R., Marek, G. W., Colaizzi, P. D., Ruthardt, B. B. & Copeland, K. S. A subsurface drip irrigation system for weighing lysimetry. *Appl. Eng. Agric.* **34**(1), 213–221, 10.13031/aea.12597 (2018).

[20] Evett, S. R. *et al*. Agronomic Calendars for the Bushland, Texas Soybean Datasets. Ag Data Commons. 10.15482/USDA.ADC/1528741 (2023).

[21] Evett, S. R. *et al*. Agronomic Calendars for the Bushland, Texas Sunflower Datasets. Ag Data Commons. 10.15482/USDA.ADC/1528067 (2022).

[22] Evett, S. R. *et al*. Agronomic Calendars for the Bushland, Texas Winter Wheat Datasets. Ag Data Commons. 10.15482/USDA.ADC/1527915 (2022).

[23] Howell, T. A., Schneider, A. D., Dusek, D. A., Marek, T. H. & Steiner, J. L. Calibration and scale performance of Bushland weighing lysimeters. *Trans. ASAE* **38**(4), 1019–1024, 10.13031/2013.27918 (1995).

[24] Evett, S. R., Colaizzi, P. D., Marek, G. W., Copeland, K. S. & Ruthardt, B. B. Analysis and quality control of weighing lysimeter water storage data. *Agricultural Water Management* **317**, 109674, 10.1016/j.agwat.2025.109674 (2025).

[25] Marek, G. W. *et al*. Post-processing techniques for reducing errors in weighing lysimeter evapotranspiration (ET) datasets. *Trans. ASABE* **17**(2), 499–515, 10.13031/trans.57.10433 (2014).

[26] Evett, S. R. *et al*. Evapotranspiration, Irrigation, Dew/frost - Water Balance Data for The Bushland, Texas Soybean Datasets. Ag Data Commons. 10.15482/USDA.ADC/1528713 (2023).

[27] Evett, S. R. *et al*. Growth and Yield Data for the Bushland, Texas Alfalfa Datasets. Ag Data Commons. 10.15482/USDA.ADC/1526355 (2022).

[28] Evett, S. R. *et al*. Growth and Yield Data for the Bushland, Texas, Soybean Datasets. Ag Data Commons. 10.15482/USDA.ADC/1528670 (2023).

[29] Evett, S. R. *et al*. Growth and Yield Data for the Bushland, Texas, Sunflower Datasets. Ag Data Commons. 10.15482/USDA.ADC/1528072 (2022).

[30] Evett, S. R. *et al*. Growth and Yield Data for the Bushland, Texas, Winter Wheat Datasets. Ag Data Commons. 10.15482/USDA.ADC/1527918 (2022).

[31] Evett, S. R. *et al*. Weighing Lysimeter Data for The Bushland, Texas Alfalfa Datasets. Ag Data Commons. 10.15482/USDA.ADC/1526357 (2022).

[32] Evett, S. R. *et al*. Weighing Lysimeter Data for The Bushland, Texas, Soybean Datasets. Ag Data Commons. 10.15482/USDA.ADC/1528684 (2023).

[33] Evett, S. R. *et al*. Weighing Lysimeter Data for The Bushland, Texas Sunflower Datasets. Ag Data Commons. 10.15482/USDA.ADC/1528074 (2022).

[34] Evett, S. R. *et al*. Weighing Lysimeter Data for The Bushland, Texas Winter Wheat Datasets. Ag Data Commons. 10.15482/USDA.ADC/1527916 (2022).

[35] Evett, S. R. *et al*. Evapotranspiration, Irrigation, Dew/frost - Water Balance Data for The Bushland, Texas Alfalfa Datasets. Ag Data Commons. 10.15482/USDA.ADC/1526370 (2022).

[36] Evett, S. R. *et al*. Evapotranspiration, Irrigation, Dew/frost - Water Balance Data for The Bushland, Texas Sunflower Datasets. Ag Data Commons. 10.15482/USDA.ADC/1528081 (2022).

[37] Evett, S. R. *et al*. Evapotranspiration, Irrigation, Dew/frost - Water Balance Data for The Bushland, Texas Winter Wheat Datasets. Ag Data Commons. 10.15482/USDA.ADC/1527917 (2022).

[38] Allen, R. G., Pereira, L. S., Raes, D. & Smith, M. Crop evapotranspiration: Guidelines for computing crop water requirements. Irrigation and Drainage Paper No. 56. Rome, Italy: United Nations FAO. (1998).

[39] Dusek, D. A., Howell, T. A. & Steiner, J. L. Evaluation of electronic temperature/relative humidity sensors. Pp. 993-999 In R.G. Allen and C.M.U. Neale (eds.) Management of Irrigation and Drainage Systems, Integrated Perspectives. *Proc. ASCE, Park City, UT. American Society of Civil Engineers,* New York, NY, 978-0-87262-919-6 (ISBN-13) | 0-87262-919-8 (ISBN-10) (1993).

[40] Evett, S. R., Tolk, J. A. & Howell, T. A. A Depth Control Stand for Improved Accuracy with the Neutron Probe. *Vadose Zone J* **2**(4), 642–649, 10.2136/vzj2003.6420 (2003).

[41] Evett, S. R., Heng, L. K., Moutonnet, P. & Nguyen, M.L. (eds.). Field Estimation of Soil Water Content: A Practical Guide to Methods, Instrumentation, and Sensor Technology. 131 pp. IAEA-TCS-30. International Atomic Energy Agency, Vienna, Austria. ISSN 1018–5518. Available at https://www.iaea.org/publications/7801/field-estimation-of-soil-water-content (2008).

[42] Evett, S. R. Some Aspects of Time Domain Reflectometry (TDR), Neutron Scattering, and Capacitance Methods of Soil Water Content Measurement. Pp. 5-49 In Comparison of soil water measurement using the neutron scattering, time domain reflectometry and capacitance methods. International Atomic Energy Agency, Vienna, Austria, IAEA-TECDOC-1137. https://www.iaea.org/publications/5932/comparison-of-soil-water-measurement-using-the-neutron-scattering-time-domain-reflectometry-and-capacitance-methods (2000).

[43] Tolk, J. A. & Evett, S. R. Lysimetry versus Neutron Moisture Meter for Evapotranspiration Determination in Four Soils. *Soil Sci. Soc. Amer. J.* **73**(5), 1693–1698, 10.2136/sssaj2009.0037 (2009).

[44] Evett, S. R., Schwartz, R. C., Howell, T. A., Baumhardt, R. L. & Copeland, K. S. Can weighing lysimeter ET represent surrounding field ET well enough to test flux station measurements of daily and sub-daily ET? *Adv. Water Resour.* **50**, 79–90, 10.1016/j.advwatres.2012.07.023 (2012).

[45] Evett, S. R. Water and Energy Balances at Soil-Plant-Atmosphere Interfaces. Pp. 127-188 In Arthur A. Warrick (ed.) The Soil Physics Companion. CRC Press LLC, Boca Raton, FL. 10.1201/9781420041651 (2002).

[46] Evett, S. R. & Steiner, J. L. Precision of neutron scattering and capacitance type soil water content gauges from field calibration. *Soil Sci. Soc. Am. J.* **59**(4), 961–968, 10.2136/sssaj1995.03615995005900040001x (1995).

[47] Evett, S. R. Soil Water Measurement by Neutron Thermalization. In B. A. Stewart and Terry A. Howell (editors). Encyclopedia of Water Science, Marcel Dekker, Inc. New York. Pp. 889-893. 2003.

[48] Evett, S.R. Soil water sensing by neutron scattering. Reference Module in Earth Systems and Environmental Sciences, Elsevier, 2022, ISBN 9780124095489, 10.1016/B978-0-12-822974-3.00046-X (2022).

[49] Hignett, C. & Evett, S. R. Neutron Thermalization. Section 3.1.3.10 In Jacob H. Dane and G. Clarke Topp (eds.) Methods of Soil Analysis. Part 4 – Physical Methods. pp. 501-521, 10.2136/sssabookser5.4.c19 (2002).

[50] Hannes, M. *et al*. A comprehensive filtering scheme for high-resolution estimation of the water balance components from high-precision lysimeters. *Hydrology and Earth System Sciences* **19**(8), 3405–3418, 10.5194/hess-19-3405-2015 (2015).

[51] Vaughan, P. J., Trout, T. J. & Ayars, J. E. A processing method for weighing lysimeter data and comparison to micrometeorological ETo predictions. *Agricultural Water Management* **88**(1–3), 141–146, 10.1016/j.agwat.2006.10.008 (2007).

[52] Savitsky, A. & Golay, M. J. E. Smoothing and differentiation of data by simplified least squares. *Anal. Chem.* **36**, 1627–1639 (1964).

[53] Brown, S. *et al*. Assessing variability of soil water balance components measured at a new lysimeter facility dedicated to the study of soil ecosystem services. *Journal of Hydrology* **603**, 127037, 10.1016/j.jhydrol.2021.127037 (2021).

[54] Peters, A. *et al*. Towards an unbiased filter routine to determine precipitation and evapotranspiration from high precision lysimeter measurements. *Journal of Hydrology* **549**, 731–740, 10.1016/j.jhydrol.2017.04.015 (2017).

[55] Peters, A., Nehls, T., Schonsky, H. & Wessolek, G. Separating precipitation and evapotranspiration from noise–a new filter routine for high-resolution lysimeter data. *Hydrology and Earth System Sciences* **18**(3), 1189–1198, 10.5194/hess-18-1189-2014 (2014).

[56] Peters, A., Nehls, T. & Wessolek, G. Improving the AWAT filter with interpolation schemes for advanced processing of high resolution data. *Hydrology and Earth System Sciences* **20**(6), 2309–2315, 10.5194/hess-20-2309-2016 (2016).

[57] Dusek, D. A. & Howell, T. A. Effects of instrument shelters on air temperature and humidity measurements. pp. 491-496. In C. R. Camp, E. J. Sadler, and R. E. Yoder (eds.) Proc. International Conference. Evapotranspiration and Irrigation Scheduling, San Antonio, TX. (1996).

[58] Howell, T. A. & Tolk, J. A. Calibration of soil heat flux transducers. *Theor Appl Climatol* **42**, 263–272, 10.1007/BF00865987 (1990).

[59] Kalu, B. A. & Fick, G. W. Morphological stage of development as a predictor of alfalfa herbage quality. *Crop Sci* **23**(6), 1167–1172, 10.2135/cropsci1983.0011183X002300060033x (1983).

[60] Berglund, D.R., McWilliams, D.A. & Endres, G.J. *Soybean Growth and Management*. NDSU Extension Service. https://www.ndsu.edu/agriculture/sites/default/files/2021-11/a1174.pdf (1999).

[61] Schneiter, A. & Miller, J. F. Description of Sunflower Growth Stages. *Crop Science* **21**, 901–903, 10.2135/cropsci1981.0011183X002100060024x (1981).

[62] Large, E. C. Growth stages in cereals illustration of the Feekes scale. *Plant Pathology* **3**, 128–129, 10.1111/j.1365-3059.1954.tb00716.x (1954).

[63] Evett, S. R. *et al*. Single- and Dual-Surface Implicit Energy Balance Solutions for Reference ET. *5th National Decennial Irrigation Conference Proceedings*, 5-8 December 2010, Phoenix Convention Center, Phoenix, Arizona USA. 10.13031/2013.35821 (2010).

[64] Evett, S. R. *et al*. Single- and Dual-Surface Iterative Energy Balance Solutions for Reference ET. *Transactions of the ASABE* **55**(2), 533–541, 10.13031/2013.41388 (2012).

[65] Lascano, R. J., van Bavel, C. H. M. & Evett, S. R. A Field Test of Recursive Calculation of Crop Evapotranspiration. *Transactions of the ASABE* **53**(4), 1117–1126, 10.13031/2013.32601 (2010).

[66] Kiraga, S., Peters, R. T., Molaei, B., Evett, S. R. & Marek, G. Reference evapotranspiration estimation using genetic algorithm-optimized machine learning models and standardized Penman–Monteith equation in a highly advective environment. *Water* **16**, 12, 10.3390/w16010012 (2023).

[67] Howell, T. A., Evett, S. R., Tolk, J. A., Copeland, K. S. & Marek, T. H. Evapotranspiration, water productivity and crop coefficients for irrigated sunflower in the U.S. Southern High Plains. *Agricultural Water Management* **162**, 33–46, 10.1016/j.agwat.2015.08.008 (2015).

[68] Thorp, K. R., Marek, G. W., DeJonge, K. C., Evett, S. R. & Lascano, R. J. Novel methodology to evaluate and compare evapotranspiration algorithms in an agroecosystem model. *Environmental Modelling & Software* **119**, 214–227, 10.1016/j.envsoft.2019.06.007 (2019).

[69] Tolk, J. A., Evett, S. R. & Howell, T. A. Advection influences on evapotranspiration of alfalfa in a semiarid climate. *Agronomy Journal*, **98**(6), 1646–1654. Portico. 10.2134/agronj2006.0031 (2006).

[70] Tolk, J. A., Howell, T. A. & Evett, S. R. Nighttime Evapotranspiration from Alfalfa and Cotton in a Semiarid Climate. **Agronomy Journal**, **98**(3), 730–736, 10.2134/agronj2005.0276 (2006).

[71] Evett, S. R., Marek, G. W., Colaizzi, P. D., Copeland, K. S.& Ruthardt, B. B. Spreadsheet for lysimeter data analysis, Bushland, Texas. Ag Data Commons. Software. 10.15482/USDA.ADC/26898151 (2024).

